# New Ways to Treat Tuberculosis Using Dendrimers as Nanocarriers

**DOI:** 10.3390/pharmaceutics10030105

**Published:** 2018-07-26

**Authors:** Serge Mignani, Rama Pati Tripathi, Liang Chen, Anne-Marie Caminade, Xiangyang Shi, Jean-Pierre Majoral

**Affiliations:** 1 Université Paris Descartes, PRES Sorbonne Paris Cité, CNRS UMR 860, Laboratoire de Chimie et de Biochimie Pharmacologiques et Toxicologique, 45, rue des Saints Peres, 75006 Paris, France; 2 CQM—Centro de Química da Madeira, MMRG, Universidade da Madeira, Campus da Penteada, 9020-105 Funchal, Portugal; 3 National Institute of Pharmaceutics and Education and Research, Raibarely 226031, India; 4 State Key Laboratory for Modification of Chemical Fibers and Polymer Materials, College of Chemistry, Chemical Engineering and Biotechnology, Donghua University, Shanghai 201620, China; liang.chen@lcc-toulouse.fr (L.C.); xshi@dhu.edu.cn (X.S.); 5 Laboratoire de Chimie de Coordination du CNRS, 205 Route de Narbonne, BP 44099, 31077 Toulouse CEDEX 4, France; anne-marie.caminade@lcc-toulouse.fr; 6 Université de Toulouse, UPS, INPT, 31077 Toulouse CEDEX 4, France

**Keywords:** tuberculosis, dendrimers, nanocarriers

## Abstract

Tuberculosis (TB) is a contagious infection that usually attacks not only the lungs, but also brain and spine. More than twenty drugs have been developed for the treatment of TB, but most of them were developed some years ago. They are used in different combinations. Isoniazid and Rifampicin are examples of the five first line TB drugs, whereas, for instance, Levofloxacin, Kanamycin and Linezolid belong to the second line drugs that are used for the treatment of drug resistant TB. Several new bicyclic nitroimidazoles (e.g., Delamanid) without mutagenic effects were developed. New TB drugs need to provide several main issues such as more effective, less toxic, and less expensive for drug resistant TB. Besides polymeric, metal-based nanoparticles, polymeric micelles and polymers, dendrimer nanostructures represent ideal delivery vehicles and offer high hopes for the future of nanomedicine. In this original review, we present and analyze the development of anti-TB drugs in combination with dendrimers. Few articles have highlighted the encapsulation of anti-TB drugs with dendrimers. Due to their unique structure, dendrimers represent attractive candidates for the encapsulation and conjugation of other anti-TB drugs presenting important drawbacks (e.g., solubility, toxicity, low bioavailability) that hinder their development, including clinic trials.

## 1. Introduction and State-of-the-Art in the Development of Nano-Based Drug Delivery of Anti-TB Drugs

Tuberculosis (TB) is an infectious disease caused by the bacillus Mycobacterium tuberculosis, also known as Koch bacillus, surviving and replicating within human alveolar macrophages and affecting other organs [1,2]. A global view of this disease can be summarized as follows: (1) according to the latest report of the World Health Organization (WHO), more than one third of the world’s population is infected with Mycobacterium tuberculosis, and approximately 2 million deaths by TB were reported in 2017; (2) current treatments—which are generally oral—are toxic to the patient; (3) TB is a common cause of death among HIV-positive patients; (4) resistant strains, such as multi-drug resistant (MDR-TB) and extensively drug resistant (XDR-TB), to all major anti-TB drugs have been observed and (5) the average cost of 6 to 32 months of treatment against TB, MDR-TB and XDR-TB is ~17,000, ~150,000 and ~300,000 US dollars, respectively [3]. Typical TB treatment follows a 6-month administration of a cocktail of four different antibiotics: rifampicin (RIF), isoniazid, pyrazinamide and ethambutol. Along with the lengthy treatment period, several side effects can occur, such as nausea, vomiting, weight loss, hepatotoxicity, adverse skin and gastrointestinal reactions and immune responses, for instance, in the case of RIF. In addition, RIF interacts with isoniazid, which is also a major anti-TB drug, leading to the corresponding insoluble hydrazine derivative [4].

**Figure 1:**
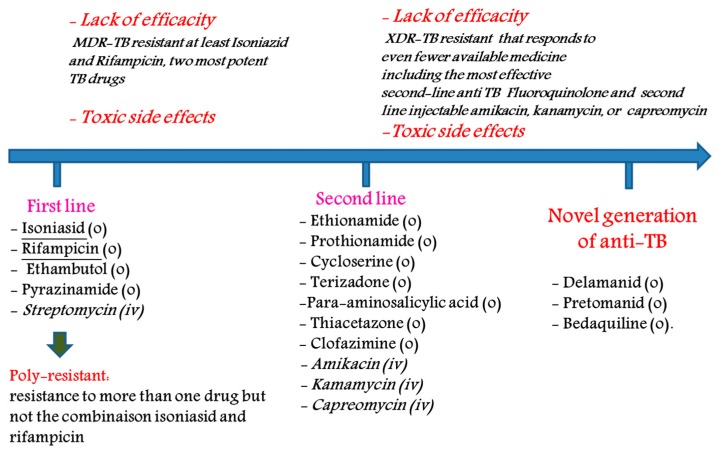
Main treatments to tackle tuberculosis (o: oral, *iv*: intravenous administration).

The main drugs used (not an exhaustive list) for the treatment of TB are as follows: (1) first-line agents: rifampicin (RIF), isoniazid (INH), pyrazinamide (PZA), thambutol (ETB) and aminoglycosides; (2) bacteriostatic second-line drugs: P-aminosalicylic acid, cycloserine and other drugs such as clofazimine, amoxicillin, clarithromycin, rifabutin and thioacetazone (Figure 1). Many of these drugs are generics [5].

**Figure 2:**
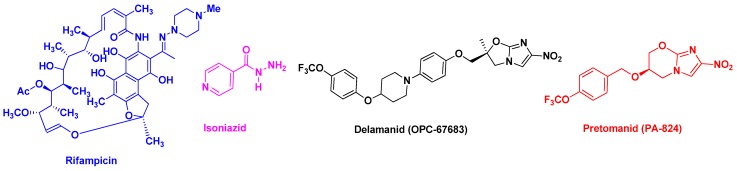
Chemical structures of Rifampicin (RIF), Isoniazid (INH), Delamanid, and Pretonamid.

There is no doubt that TB remains a major global health problem, and new treatments to combat the crisis of drug resistance in TB are urgently needed. To this end, several new bicyclic nitroimidazoles without mutagenic effects were developed, such as OPC-67683 (delamanid, Otsuka Pharmaceuticals) and PA-824 (pretonamid, PathoGenesis) (Figure 2). Delamanid was approved by the European Medicines Agency (EMA) in 2014 for pulmonary multi drug-resistant TB in adult patients [6,7], whereas pretonamid (Figure 2) is currently in Phase II/III clinical trials for the treatment of TB. Recently, the Global Alliance for TB drug development and the University of Auckland advanced TBA-6354 to Phase I, but the drug showed neurotoxicity in healthy volunteers [8,9]. In 2012, the FDA granted accelerated approval to the diarylquinonoline derivative, SirturoTM (bedaquiline, TMC-2017) for treatment in adults of multidrug-resistant tuberculosis. It is the first new anti-TB drug to be approved after 1998 (Rifapentine) [10]. This drug was the first to be introduced specifically for the treatment of multidrug-resistant tuberculosis in combination with other drugs such as Delamanid combination for the treatment of patients with pulmonary extensively drug-resistant tuberculosis [11].

Nanotechnology represents a multidisciplinary field encompassing various domains such as chemistry, materials, biology, physics, diagnosis and engineering, including the synthesis of nanodevices. The pharmaceutical industry adopts nanotechnology throughout the R&D process [12]. Thus, nanoformulations and nanocarriers have made a significant impact on the delivery of therapeutic agents and diagnostics through the development of several nanodevices such as liposomes, nanocrystals, nanoparticles and dendrimers. These nanodelivery platforms can improve the solubility and, consequently, the bioavailability and pharmacokinetic/pharmacodynamic (PK/PD) ratio, reduce the therapeutic dosage (by increasing the drug exposure level over a long period of time) and minimize off-target effects (adverse side effects) of carried drugs such as small molecules, macrocycles and peptides. Finally, the use of nanoparticles can simplify the treatment versus, for example, drug combinations [13].

These therapeutic agents can be encapsulated within nanoparticles, conjugated to them or complexed on their surface. Genes and vaccines can also be carried. In addition, many efforts have been devoted to developing targeted nanoparticles to deliver therapeutic agents to the right tissues (e.g., brain, kidney, lung), cells (e.g., tumor cells) and inside cell compartments (e.g., nucleus, mitochondria, cytosol) [14].

A global view of the modulation of several physicochemical properties has been proposed by Choi, named ‘Choi criteria’ [15]. Composition of the nanoparticles manage their biodegradation and toxicity effects, surface properties control their targeting and biodistribution properties, whereas size and shape govern their excretion and clearance profiles.

**Figure 3:**
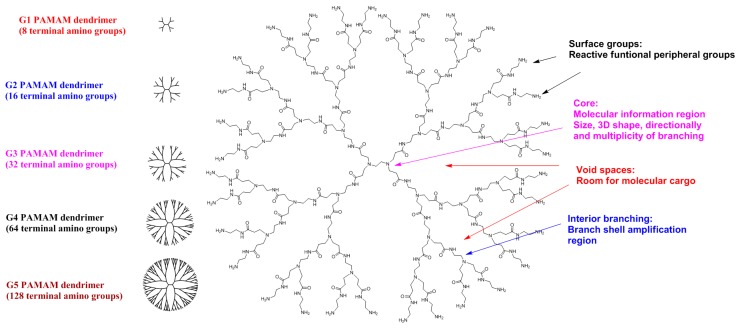
Schematic 2D representation of G3 PAMAM dendrimer as a typical dendritic structure.

Besides polymeric, metal-based nanoparticles, polymeric micelles and linear polymers, dendrimer and dendron nanostructures represent ideal delivery vehicles and offer high hopes for the future of nanomedicine [16]. Dendrimers (from the Greek words “dendros” and “meros”) are a family of nanosized macromolecules characterized by a highly homostructural, branched three-dimensional (3D) architecture and compact, spherical geometry in solution. Dendrimers represent globular macromolecules, with a highly branched 3D architecture, whose shape and size can be precisely controlled. They display an exponential number of dendritic branches (hydrophobic and hydrophilic moieties) radiating from a central core. The dendrimer diameter increases linearly, while the number of surface groups increases exponentially with each generation. Low-generation dendrimers are usually flexible, while higher generation compounds are denser and increasingly rigid [17]. A schematic of a typical dendritic structure for biomedical applications is illustrated in Figure 3.

The dendritic macromolecular structure can be divided into four main components: (a) a central core moiety; (b) interior layers (generations, Gn, where n is 0, 0.5, 1, 1.5, …) made of regularly repeating branching units attached to the core; (c) terminal functionalities distributed in a 3D space; (d) void spaces, which are rooms for molecular cargo, such as anti-cancer agents [18]. The main dendrimer types used are PAMAM dendrimers (Starbust^®^), poly-etherhydroxyl-amine (PEHAM) dendrimers (Priostar^®^), PPI dendrimers (Astramol^®^) carbosilane dendrimers, and phosphorus-based dendrimers developed by J-P. Majoral and A-M. Caminade [19].

In 2012, Starpharma initiated two pivotal Phase III trials for the treatment of bacterial vaginosis with VivaGel^®^ (SPL7013 Gel) [20]. This active polyanionic G4-poly(*l*-lysine)-type dendrimer has 32 naphthalene disulphonate groups on the surface and showed potent topical vaginal microbicidal activity. Starpharma has now received Food and Drug Administration Phase III approval. Starpharma/AstraZeneca has recently advanced from Phase I to Phase II with a poly(lysine)-dendrimer-based nanocarrier encapsulating docetaxel (DEP^®^ docetaxel), which shows superior anticancer activity against several solid cancer types, including breast, prostate, lung and ovarian tumors [21].

With the aim of improving the bioavailability of anti-TB drugs in general and RIF in particular, several studies have been developed using drug delivery system approaches (nanoformulations) such as (1) nanodispersions including nanosuspensions, nanoemulsions, solid dispersions and niosomes; (2) polymeric and non-polymeric nanoparticles; (3) polymeric micelles and analogues and (4) liposomes and dendrimers (vide infra). The main anti-TB drugs carried by liposomes are streptomycin, gentamycin, sparfloxacin, amikacin, clofazimine, INH, RIF, PZA, rifabutin (RFB) and capreomycin; by niosomes, RIF and by nanoparticles and microparticles, INH, RIF, PYZ, ETB, streptomycin, moxifloxacin, PZA, ETB and econazole [22].

In this original review, we present and analyze the development of anti-TB drugs in combination with dendrimers. To our knowledge, there have been few articles featuring this nanoformulation. Importantly, similar to the development of nanocarriers in the oncology domain, lodging of *M. tuberculosis* strains in the phagosome compartment of macrophages with an acidic pH (~4.5) allows the strategy of developing nanocarriers, such as tunable dendrimers, as a pH-based vectorization system that retains the anti-TB drugs in the blood (pH 7.4) and effectively release them in an acidic environment. Due to their unique structure and diverse route of administration, dendrimers represent attractive candidates for the encapsulation and conjugation of anti-TB drugs presenting important drawbacks (e.g., solubility, toxicity, low bioavailability) that hinder their development, including clinic trials.

## 2. Encapsulation of Anti-TB Drugs within Dendrimers

**Figure 4:**
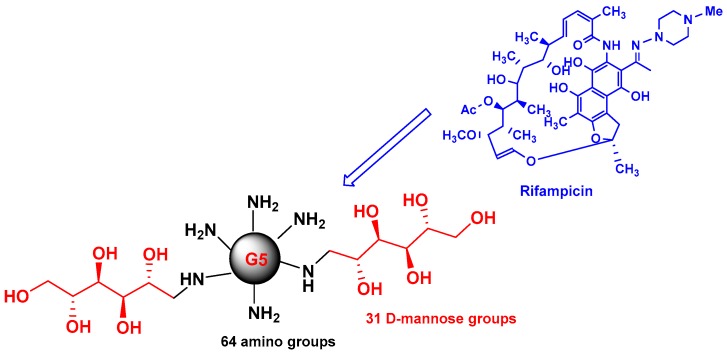
Schematic 2D of mannosylated G5 EDA-PPI dendrimer and RIF Me.

Early studies were performed by Palanirajan Vijayaraj Kumar and co-workers on the development of mannosylated dendritic architectures (G5 EDA-PPI dendrimers) for the selective delivery of RIF to alveolar macrophages [23]. The mannosylated G5 EDA-PPI dendrimer is a fifth-generation poly(propyleneimine) (PPI, 64 amino groups on the surface) dendrimer with an ethylene diamine (EDA) core and grafted on the surface with ~30 D-mannose groups (Figure 4). RIF is an essential component of the cocktail of anti-TB combination drugs, as shown in Figure 1. The mechanism of action of RIF is related to the inhibition of the subunit of the bacterial RNA polymerase, which inhibits gene transcription. It is emphasized that several side effects of RIF are related to its poor pharmacokinetic profile, mainly due to its low solubility in water. In addition, under gastric conditions (pH 4–5), RIF is hydrolyzed into the less soluble compound 3-formyl-rifampicin. Mannose was selected because this sugar molecule is recognizable by lectin receptors on the surface of phagocytic cells and consequently improves the uptake of nanocarriers bearing mannose on their surface and drugs in their void spaces by the cells of the immune system.

Using the very well-known dissolution technique, about 37 RIF have been incorporated into PPI dendrimers. SEM studies showed an irregular shape and agglomerated mannosylated dendrimers with a median diameter less than 5 μm. Differential scanning calorimetry studies suggested that loaded mannosylated PPI dendrimers did not form a physical mixture. The solubility of the nanodevice obtained by the incorporation of RIF within the PPI dendrimer is ~50 mg/mL, while a decrease in solubility was observed when RIF was encapsulated in a mannosylated dendrimer (~5 mg/mL), but this solubility level is twice that of the aqueous solubility of RIF alone. Non-covalent interactions between RIF and the mannosylated dendrimer were observed. Both hydrogen bonding and hydrophobic interactions (~37%) with the core were observed.

Hemolytic toxicity of the G5 EDA-PPI mannosylated dendrimer was evaluated against red blood cells emphasizing the significant decrease in toxicity of mannosylated versus non-mannosylated dendrimers (2.8% versus 15.6%) due to inhibition of the interaction of the charged quaternary ammonium ion with cells. The high hemolytic toxicity of unmodified PPI dendrimers, with all the surface bearing amino groups, precludes their clinical applications. Similarly, PAMAM dendrimers must be modified on their surface by the introduction of groups that decrease the cationic surface aspect and, consequently, toxicity. Mannosylated dendrimers displayed negligible cytotoxicity against Vero cells at a concentration of 100 μg/mL, as did the RIF-loaded mannosylated dendrimer (~85% viability). In the same assay, the group of RIF alone showed a viability of ~50% at the same concentration.

The authors mentioned that RIF remained for a long duration (~120 h) in the interior cavities of mannosylated dendrimers at physiological pH of 7.4 versus <10 h with the PPI dendrimer. Importantly, a high drug release rate was observed at pH 5, which simulates the pH of phagolysosomes. A clear increase (intracellular concentration) in the cellular uptake of RIF-loaded mannosylated PPI dendrimers by alveolar macrophages (AM) from rat lungs versus RIF alone was observed. A quite linear curve amount of RIF incorporated in AM versus the amount of RIF was observed. Consequently, the mannosylated G5 EDA-PPI dendrimer represents a good nanocarrier system for the delivery of hydrophobic drugs such as RIF in the tuberculosis domain. 

Using the same approach, the authors described similar effects using 4G and 5G PEGylated PPI dendrimers for the sustained delivery of RIF [24]. The grafting of the PEG chains on the surface of 4G and 5G PPI dendrimers strongly increased their drug entrapment capability from 28% and 39% to 57% and 61% for G4 and G5 dendrimers, respectively. Also, the introduction of PEG chains improved the control of release of RIF after 36 h, with a cumulative release of 97.3% and 46.3% for G4 and G5 dendrimers, respectively. In addition, low and high hemolytic toxicities were observed (1–3%) for PEGylated PPI dendrimers and non-pegylated PPI dendrimers, respectively, with the same generations (14–20%). 

Bellini and co-workers described molecular dynamics (MD) simulation studies of the association of the anti-tuberculosis drug rifampicin (RIF) with the G4-PAMAM dendrimer [25]. It is well known that molecular dynamics (MD) simulation represents a powerful tool to study several types of molecular systems, such as association systems. This study showed that, experimentally, the maximum number of RIF molecules loaded is 20 RIF molecules per G4-PAMAM dendrimer. This result is concordant with the work of Kumar et al. emphasizing the association of a maximum of ~37 RIF molecules per mannosylated G5-PPI dendrimer (vide supra). 

In addition, the authors studied the drugs occupied volumes of RIF and that of a similar compound ibuprofen, which together belong to the class II biopharmaceutical classification system (BCS) as defined by the FDA, which is characterized by low solubility and high permeability [26]. The maximum number of ibuprofen molecules is 78 per dendrimer, approximately four times higher than the number of RIF per G4 PAMAM dendrimer. It is important to note that the molecular weight of RIF is approximately four times higher than that of ibuprofen (MW 822 versus 206), and RIF is approximately four times larger than ibuprofen (1.086 versus 0.276 nm^3^).

Based on the docking of RIF molecules within the G4 PAMAM cavities (20 RIF molecules per dendrimer determined experimentally, vide supra), molecular dynamic simulations were generated at neutral and low pH. Reasonable stability of the complex at neutral pH was observed, whereas at low pH, RIF molecules were rapidly and simultaneously expelled to the solvent bulk. This result underlines the potential role of PAMAM dendrimers as nanocarriers to deliver drugs in acidic cellular structures such as alveolar macrophages. 

**Figure 5:**
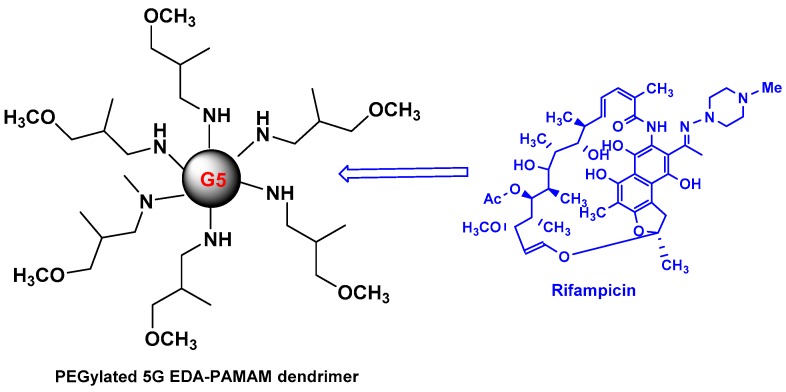
PEGylated 5G EDA-PAMAM dendrimers and RIF.

Recently, P. Dineshkumar and co-workers emphasized the development of RIF loaded PEGylated 5G EDA-PAMAM dendrimers [27] (Figure 5). The PEGylation of 5G PAMAM dendrimers was confirmed by Fourier Transform Infrared Spectrophotometry (FTIR) technique, and ^1^H-NMR spectra. The RIF entrapment efficiency in PEGylated dendrimer was found to be ~99%. The drug release rate of RIF is 81% and 98% for PEGylated (in 120 h) and non-PEGylated (in 72 h) PAMAM dendrimers, respectively. The hemolytic studies showed that non-PEGylated PAMAM dendrimer revealed 11.6 to 25.3% of toxicity, whereas the PEGylated PAMAM dendrimer strongly showed lower toxicity effects (less than 2.5%) due to the inhibition of interaction of red blood cells with quaternary ammonium groups on the surface of dendrimers. Similar effects were highlighted in early studies with dendrimers bearing cationic groups on their surface [28].

Interestingly, in vivo studies were performed in Wistar albino rats, and revealed interesting pharmacokinetic (PK) parameters of PEGylated 5G EDA-PAMAM dendrimers loaded RIF versus RIF alone [29]. Table 1 highlights the PK profile of 5G EDA-PAMAM dendrimers loaded RIF and RIF (0.5–120 h). After 6 h there was no presence of RIF in plasma, whereas in 5G EDA-PAMAM dendrimers, RIF remained for 120 h. Low and prolonged release of RIF were observed inducing higher area under the plasma drug concentration-time curve (AUC), half-life (t_1/2_) and mean residence time (MRT) values for PEGylated 5G EDA-PAMAM dendrimers loaded RIF versus free RIF. No change either in appearance or in RIF release from 5G EDA-PAMAM dendrimers loaded with RIF were observed after 3 months (~40 °C) storage.

**Figure 6:**
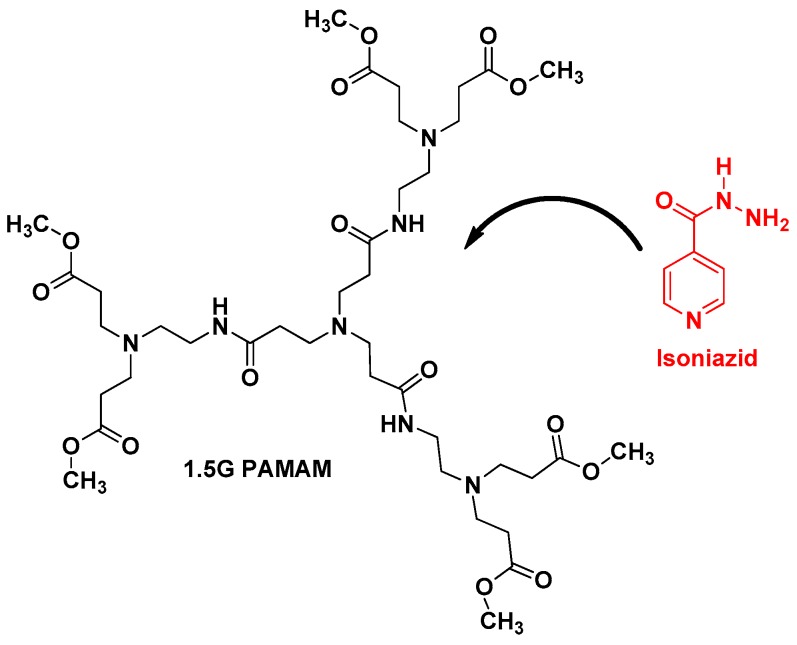
Chemical structure of 1.5G PAMAM dendrimer and Isoniazid (IND).

Another very important anti-TB drug has been also loaded with dendrimers, isoniazid (IND) [28]. N. Singh et al. described the loading of IND with 1.5G PAMAM dendrimer using dialysis method (Figure 6). UV spectroscopy and FTIR techniques confirmed the loading of IND. This formulation revealed that around 93.25% of IND was constantly released up to 24 h. In addition, drug release kinetic studies demonstrated that the kinetic is zero order corresponding to a non-Fickian diffusion probably due to the sharp boundary separating the void spaces, where IND is encapsulated, and the surface of the dendrimer [30].

## 3. Concluding Remarks and Perspectives for Clinical Translation

The chronic pulmonary infectious disease TB is the second most deadly infectious disease, causing approximately 2 million deaths per year. Despite curative treatments that have been available for over 50 years, the treatment is complex and lasts for up to 9 months. Unfortunately, anti-TB drugs revealed side effects and adverse events associated with medications inducing, for instance, a change in the dosage of drugs, discontinuation of treatment and additional treatment. Several complications increased morbidity and mortality. Very importantly, to increase treatment efficacy, including avoiding resistance (MDR-TB and XDR-TB), it is necessary to utilize multidrug regimens, which have also been associated with an increased incidence of side effects. Consequently, the risks of treatment failure and relapses are higher. Clearly, monitoring treatment and developing new strategies will be crucial to decreasing the cost of treatment and the incidence of serious drug-related adverse effects. In this direction, the development of effective nanotechnology represents a very important domain in the TB field. Several techniques, such as nanodispersions, polymeric nanoparticles, polymeric micelles, liposomes and dendrimers, have been used. 

Dendrimers have a polymeric architecture with tunable defined structures and represent versatile drug delivery systems. Thus, in the dendrimer domain, few articles have highlighted the encapsulation of anti-TB drugs such as RIF (vide supra). No conjugation of anti-TB drugs was described to date. Extensive application of dendrimers as nano carriers has been described essentially in the oncology domain.

We believe strongly that a new avenue has appeared in the development of biocompatible dendrimers as nanocarriers in the TB domain. Dendrimers can potentially entrap and/or conjugate high-molecular-weight hydrophilic/hydrophobic chemical entities or a combination of chemical entities, which generally suffer from various limitations like low aqueous solubility and short half-lives, by host-guest interactions and covalent bonding (a prodrug approach), respectively. Consequently, it is necessary to design new biodegradable, biocompatible, water-soluble and nontoxic dendrimers which can deliver a drug or a mixture of drugs, such as anti-TB drugs, efficiently through an endocytosis process to improve treatment efficacy and, consequently, patient compliance. One other advantage of dendrimers is their use in both therapeutic and diagnostic applications, and they represent a new generation of nanoparticle-based therapies. Regarding the toxicity of dendrimers, in vitro and in vivo studies are crucial to evaluating cell viability, hematological toxicity, immunogenicity, biocompatibility and biodistribution, to establish risk/benefit ratios and to assure their clinical development. Importantly, Good Manufacturing Practice (cGMP) requirements are indispensable for Investigational New Drug approval, to ensure the quality, reproducibility and reliability of in vitro and in vivo data and to secure successful development.

The successful translation from research to the clinic should be performed by the development of such biocompatible dendrimers to jump the ‘valley of death’ successfully. To this end, several of us (S.M., J.-P.M., X.S.) recently proposed a simple set of guidelines to move towards an Investigational New Drug application [31].

## Figures and Tables

**Table 1:** Pharmacokinetic (PK) profile of 5G EDA-PAMAM dendrimers loaded rifampicin (RIF) and RIF.

Main PK Parameters	RIF	5G EDA-PAMAM Dendrimers Loaded RIF
*C*_max_ (µg/mL)	19.81	47.85
*T*_max_ (h)	2	48
AUC (µg/L*h)	1154	71451
t_1/2_ (h)	2.14	66.3
MRT (h)	4.01	90.18
